# Unmodified starch as water-soluble binding polymer for chromium ions removal via polymer enhanced ultrafiltration system

**DOI:** 10.1186/2052-336X-12-61

**Published:** 2014-03-11

**Authors:** Nurul Huda Baharuddin, Nik Meriam Nik Sulaiman, Mohamed Kheireddine Aroua

**Affiliations:** 1 Department of Chemical Engineering, University of Malaya, Kuala Lumpur, Malaysia; 2 School of Environmental Engineering, Universiti Malaysia Perlis, Perlis, Malaysia

**Keywords:** Polymer-enhanced ultrafiltration, Unmodified starch, Polyethylene glycol, Metal ions removal, Complexation

## Abstract

**Background:**

In this study the removal of Chromium (III) and Chromium (VI) ions are investigated via polymer enhanced ultrafiltration under important process parameters. This study proposes the use of unmodified starch as a novel polymer in the ultrafiltration process and its performance on the removal of chromium ions was compared with a commonly used polymer, polyethylene glycol.

**Methods:**

The experiments were carried out at 1.5 bar and different pH values by using 10 kDa hollow fiber membrane operating in a cross-flow mode.

**Results:**

The best chromium ions removal obtained approached 99% for Chromium (III) ion by unmodified starch at alkaline pH region and at pH 7 for Chromium (VI) ions retention by polyethylene glycol. Permeate flux behavior are fluctuated for both chromium ions tested at high metal ion concentrations. Low concentration of unmodified starch is applied to reduce gelatinization behavior.

**Conclusions:**

The findings suggest that binding of chromium ions by unmodified starch is related to granule structure which is probably a principal indicator of the non-ionic behavior of unmodified starch.

## Background

Recovery of metal ions from valuable metals discharge by industrial or domestic effluents is well practiced through the separations technique for dilute or concentrated solutions for the past few years [1]. The major species of heavy metals that cause chronic disorders to organisms are chromium, copper and zinc; these disorders can occur through ingestion if taken accidentally at limits beyond acceptable to human bodies [2]. In the natural environment, most chromium species are present in oxidation ranges of -2 to +6 [3]. These chromium species such as chromate (CrO_4_^2-^), bichromate (HCrO_4_^2-^) and dichromate (Cr_2_O_7_^2-^) exert toxic and hazardous pressure on the ecosystem as they are pervasive only on the subsurface environment and do not effectively disintegrate into the soil in alkaline and less acidic conditions [4].

In order to address this problem, current technology has come up with an alternative, which is Polymer Enhanced Ultrafiltration (PEUF), described previously [5-7]. In this process, adsorptive mechanism of polymers efficiently bound with metal ions form molecular complexation and through the ultrafiltration process, this macromolecular is rejected. A diluted permeate that can be discharged into the sewage or employed for a specific purpose is thus obtained [8]. In our study, a water soluble polymer, namely unmodified starch and polyethylene glycol (PEG) which mostly has no negative impact on our environment were used.

The unique criteria of unmodified starch are that it is an inexpensive agricultural material and is environmental friendly; these are the reasons for introducing this polymer into the PEUF system. Although it is preferable to modify starch to improve its end-use properties, it can even be used without modification in the separation process. Hence, unmodified starch was proposed in this study for complexation of ultrafiltration system towards the metal ion-polymer interaction.

There has been only limited number of studies on cation binding by starch in the previous decade. Hollo et al. suggested that cation binding was related to phosphate content of starch [9]. Wettstein et al. showed that divalent cations were bound by cross linked starch phosphate where selectivity increased in the order Ca < Ni < Zn < Cu [10]. One of the most important finding has been that the adsorptive affinity of starch towards alkaline metals does not markedly affect the species of starch, content of linear fraction, granule size or micellar organization within the granule [11].

The other polymer used in this study is commonly used water-soluble polymer, PEG. In PEUF studies, one of the most important operating parameters is pH. As molecules of metal are able to form complexation of metal hydroxyl that could increase to sizes greater than membrane pores at high pH region, the latter is effectively rejected, particularly for Zn (II) [12]. The implications of this finding is that at certain pH range and metal ion concentration, there is a high possibility of achieving great retention of metal ions-polymer as well as the type of behavior between metal ions-polymer used in experimental works.

Hence, the objective of this study is to investigate the effects of operating parameters including pH, heavy metal feed concentration and polymer concentrations on the retention and permeate flux of heavy metal ions namely Cr (III) and Cr (VI). The comparisons on the performance of selected polymers, namely unmodified starch and PEG is carried out during the ultrafiltration process for efficient study of chromium ion removal.

## Methods

### Materials

The experiment was carried out using unmodified starch in powder form and polyethylene glycol (PEG 200) as selected water soluble polymer in the form of solutions (analytical grade) from ACROS Organics, along with sodium hydroxide and hydrochloric acid for pH adjustment. 1000 mg/l of Cr (III) and Cr (VI) ions were used as metal solutions. Cr (VI) was prepared from potassium dichromate and Cr (III) solutions from Chromium (III) nitrate hexahydrate. All chemicals were used from ACROS Organics without treatment and proved satisfactory as an analytical grade. For the purpose of dilution and feed solution preparation, ultrapure water obtained using Millipore water purification system was used. The ultrafiltration system carried out consisted of a membrane system 10,000 Da cut off from GE Healthcare (Model: UFP-10-C-MM06A) with an effective filtration area of 26 cm^2^ and a pure water permeate flux, Jw, of 0.9421 cm/min after 20 min of operation at 1.5 bar, equipped with polysulfone hollow fiber membrane.

### Complexation-ultrafiltration apparatus

The 250 cm^3^ of Cr (III) and Cr (VI) sample solutions containing different concentrations of metal and selected water soluble polymers (unmodified starch and PEG) were stirred for complexation of metal-polymer. The prepared solutions were then placed in beaker and the ultrafiltration process was started. Fixed pressure of 1.5 bar was applied with a permeate water flux, J_w_ 0.6982 cm/min after 20 minutes of running the experimental work. The membrane used, as noted earlier, was polysulfone hollow fiber with effective filtration area of 26 cm^2^.

The ultrafiltration experiments started after both the retentate and permeate solutions were cycled continuously for about 10 minutes to ensure that the solutions achieve stable condition. At constant pH value, samples began circulating after 10 minutes and the experiment was run continuously for 2 hours. The feed, retentate and permeate solutions were then collected separately in different containers and stored in the refrigerator with temperature kept at a constant 4°C. Sample solutions were analyzed using the Inductivity Couple Plasma (ICP Optical Emission Spectrometer Optima 7000DV) for metal ions solution concentrations especially for retention analysis. The membrane needed to be cleaned with ultrapure water immediately after the UF process and rinsing was continued with 0.1 M of NaOH and 0.1 M NaOCl. Water flux readings had to be recorded each time before the experiment began to ensure cleanliness of the membrane.

### Permeate flux and metal ions retention

After the experiments had been carried out for 2 hours at the transmembrane pressure (TMP) 1.5 bar, permeate solutions were collected. The permeate flux was determined from the following equation in cm^3^/(cm^2^/min):

(1) $$\mathrm{Flux};F=\frac{\Delta V}{\left(A,\Delta t\right)}$$

where △V is the volume of permeate, A is the effective membrane area, and △t is the sampling time. Retention values were calculated from the formula:

(2) $$\mathrm{Retention},R=1-C_{p}/C_{f}$$

where C_p_ is the concentration of metal ion in permeate and C_f_ is the concentration of metal ion in the feed. C_p_ in this experiment obtained average metal ions throughout the UF system [5-7,13]. All experiments were carried out twice and no significant differences were observed between the 2 runs.

### Chemical analysis

Sample solutions (feed, retentate and permeate solutions) were analyzed using the Inductivity Couple Plasma (ICP Optical Emission Spectrometer Optima 7000DV). The purpose was to analyze metal ions solution concentrations, especially for retention analysis.

## Results and discussion

### Potentimetric titration studies

Relative selectivity for metal ions depends on the overall basicity of the compounds where the acid-base behavior was potentiometrically studied. Effects of pH on the electrode potential at various selected metal ions concentration Cr (III) and Cr (VI) were studied by adding diluted solutions of 1 M HCl or 1 M NaOH. The strength of an acid or base determines the sharpness of the change. Based on the acid-base titration curve in potentiometry study, the influence of pH and ionic strength on polymeric molecule performance is clearly demonstrated on reaction of selected polymers (unmodified starch and PEG with and without presence of Cr (III) and Cr (VI)).

### Potentiometric titration study on chromium (III)

**Figure 1:**
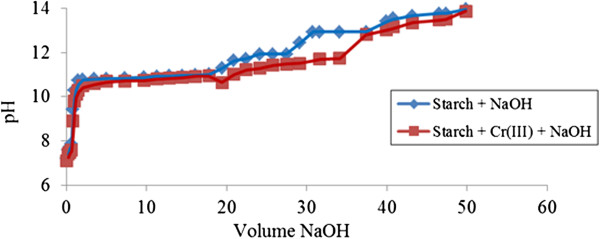
**Acid-base titration with NaOH of unmodified starch 0.05% (w/v) in presence of Cr (III).**

The titration curve (V_NaOH_, pH) during neutralization of unmodified starch by sodium hydroxide in the presence of Cr (III) ions is revealed in (Figure 1). There is no complexation between unmodified starch and Cr (III) at the early stages of titration since the curve of unmodified starch by itself and in the presence of Cr (III) showed no gaps at all. Complexation began to occur at pH > 10 as the curve between these two species, i.e. unmodified starch by itself and in the presence of Cr (III) appears to have more gaps as well as wider gaps at pH 11-12.

This is mostly due to unmodified starch which is able to adsorb Cr (III) ions at this pH region. This phenomenon is demonstrated in (Figure 1) where the pH remains lower whenever interaction between ‘unmodified starch-Cr (III)’ takes place extensively as the Cr (III) ions are capable of filling in the available space for unmodified starch molecular granules. This attracting process between Cr (III) and unmodified starch begins to retard when most of the empty spaces of the unmodified starch have been filled with Cr (III) ions at pH above 13.

**Figure 2:**
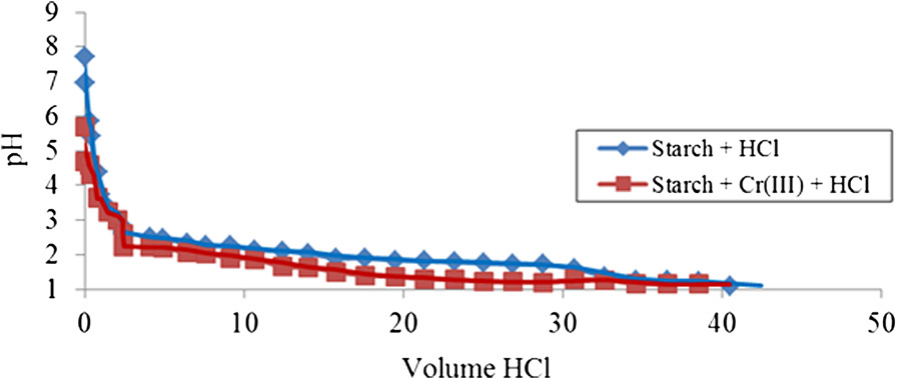
**Acid-base titration with HCl of unmodified starch 0.05% (w/v) in presence of Cr (III).**

**Figure 3:**
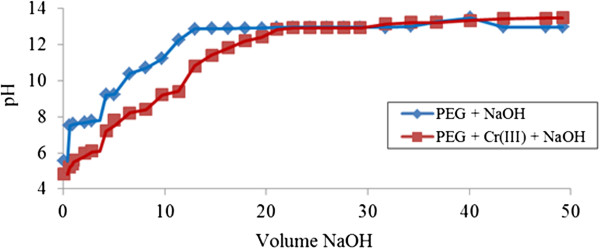
**Acid-base titration with NaOH of PEG 1.0% (v/v) in presence of Cr (III).**

Similar behavior was obtained in the case of neutralization of unmodified starch by hydrochloric acid in the presence of Cr (III) ions as demonstrated in the curves (V_HCl_, pH) presented in (Figure 2). Where neutralization of unmodified starch with hydrochloric acid in the presence of Cr (III) is concerned, the complexation occurs when the pH point reaches pH 2 where smaller gaps appear. Attractive adsorption of Cr (III) ions by unmodified starch is occurring can be observed in the curve of unmodified starch with the presence of Cr (III) ions starting to distance. This implication is due to the capability of unmodified starch approaching Cr (III) ions to fill its empty sites to form macromolecules due to the unique characteristic of unmodified starch which has neutral pH 7. The adsorption process is retarded when the region is too acidic, demonstrated by overlapping curve within unmodified starch in the presence of Cr (III) at pH near 1.

(Figure 3) shows the dramatic shift of PEG neutralization curve when Cr (III) is present in the solution. The shift in the neutralization curve indicates the fast interaction of adsorption process occurring from the beginning of titration (pH <6). More gaps appear at higher pH values, indicating the presence of greater adsorption for natural PEG to provide an available site for Cr (III) ions to be attracted to form macromolecule complexes. Although the adsorption process occurs effectively over most of the pH range, at one point when pH exceeds 13, the adsorptive mechanism between ‘Cr (III)-PEG’ becomes ‘reluctant’.

### Potentiometric titration study on chromium (VI)

**Figure 4:**
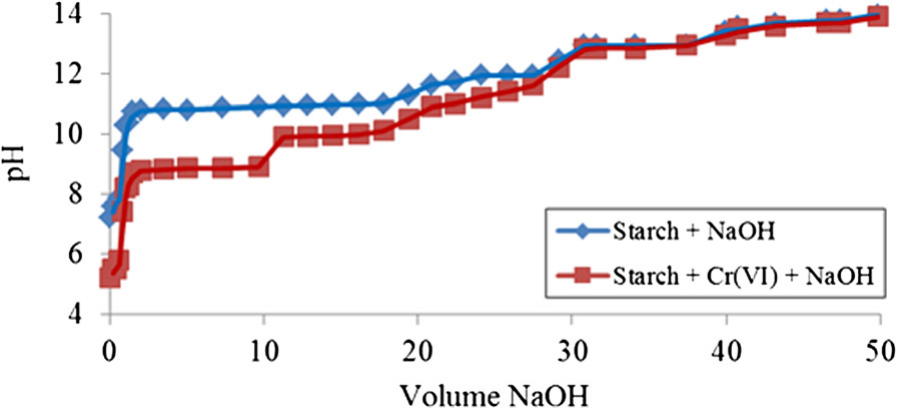
**Acid-base titration with NaOH of unmodified starch 0.05% (w/v) in the presence of Cr (VI).**

The titration curve (V_NaOH_, pH) during neutralization of unmodified starch by sodium hydroxide in the presence of Cr (VI) ions is presented in (Figure 4). Complexation occurs from the beginning of titration (pH > 5) due to the dissimilar pattern of neutralization curves between unmodified starch by itself and in the presence of Cr (VI) ions. Wider gaps appear at pH above 9, indicating a greater adsorption mechanism whereby Cr (VI) is able to penetrate the surface of unmodified starch extensively. The pH remains lower during ‘Cr (VI)-unmodified starch’ titration due to the occurrence of higher interaction compared to neutralization of unmodified starch alone. The gaps demonstrated are smaller at pH above 10 in the case of unmodified starch itself indicating adsorption process is over when the pH is above 12.

**Figure 5:**
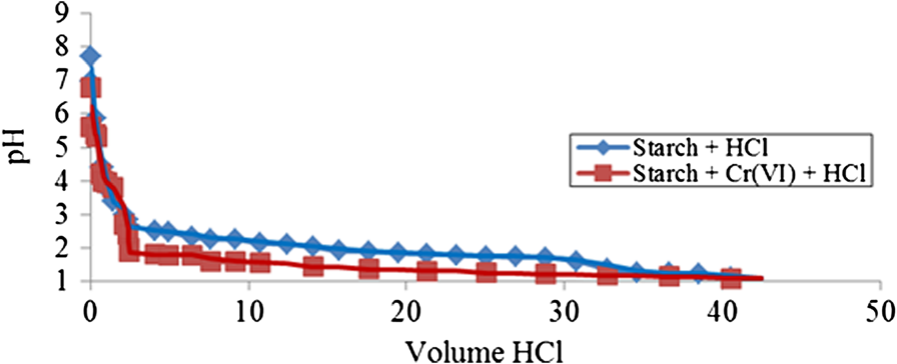
**Acid-base titration with HCl of unmodified starch 0.05% (w/v) in presence of Cr (VI).**

In the case of neutralization of unmodified starch in the presence of Cr (VI) ions, a different behavior was observed when hydrochloric acid was used as indicated by the curves (V_HCl_, pH) presented in (Figure 5). For neutralization of unmodified starch with Cr (VI) ions by HCl, at the initial titration process, the curve follows a similar trend as that for neutralization of unmodified starch by itself and is not affected by the presence of Cr (VI) ions. This indicates that in this pH range (pH > 3), no interaction between ‘Cr (VI)-unmodified starch’ was formed. At lower pH values, modifications appeared to indicate that the Cr (VI) ions are able to fill in the available sites of unmodified starch through adsorption process. At this point, the remaining pH during titration is observed to be lower due to effective interactions occurring compared to that of unmodified starch alone.

**Figure 6:**
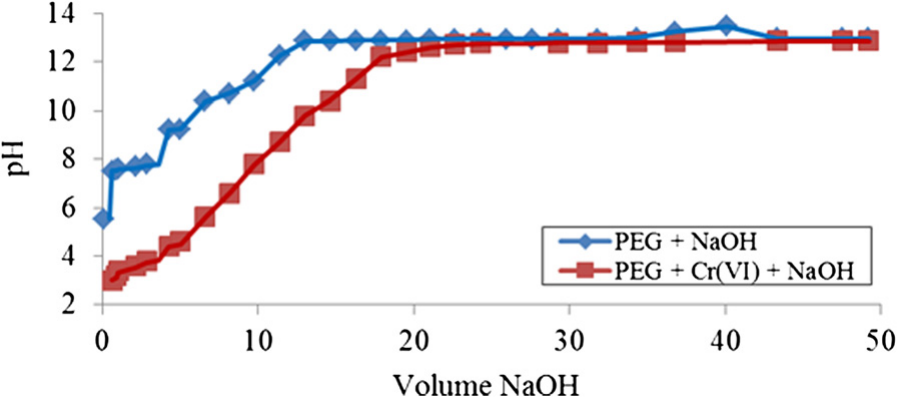
**Acid-base titration with NaOH of PEG 1.0% (v/v) in presence of Cr (VI).**

The titration curve (V_NaOH_, pH) during neutralization of PEG by sodium hydroxide in the presence of Cr (VI) ions is presented in (Figure 6). Complexation occurs from the beginning of titration (pH > 3) due to larger gaps appearing between the neutralization curve of PEG by itself and in the presence of Cr (VI) ions. These wider gaps of neutralization remain probably due to the high interactions of Cr (VI) ions attracting to provide space on unmodified starch molecular surface and it happens at most pH regions probably forming larger molecules. This extensive adsorption mechanism is observed until the pH is above 12. Slower interactions take place after that until the point where no effect of Cr (VI) ions presence in the neutralization with unmodified starch is obtained, i.e. at pH over 13.

### Effect of pH on chromium ions retention by PEUF

#### Effect of pH on chromium ions retention using unmodified starch

**Figure 7:**
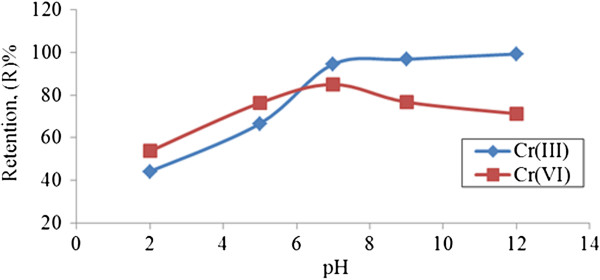
**Effect of pH chromium ions retention using 0.05% (w/v) unmodified starch.** (p = 1.5 bar, flowrate = 115 cm/min).

(Figure 7) shows the chromium ion retention as function of pH at 10 mg/l of initial chromium concentrations. In the cross flow results, low retention of complexation reaction is found at low pH range. In contrast, retention coefficient of Cr (III) and Cr (VI) approached 94.5% and 84.9%, respectively for the complexation at pH = 7, which means that chromium ions were efficiently removed from the affluent at optimum working conditions. This is most probably due to the physical adsorptive mechanism of unmodified starch containing amylopectin which has highly branched molecular structures that allow the penetration of chromium ions at the molecular surface [13]. The removal of Cr (III) by unmodified starch at optimum working conditions meets the Department of Environmental (DOE) permissible limits of effluent discharge standards of 1 mg/l [14]. Using starch without modification is found to be efficient in removal Cr (VI) ions without reducing to Cr (III). In consequence, it can also avoid precipitation.

There is a high possibility of bonding occurring between unmodified starch and chromium ions via chemical interaction since starch is also able to have pendant hydroxyl (OH) groups capable of forming hydrogen bonds H^+^ at position 2-, 3- and 6- in glucose to adsorb the metal cations. This possibility can be expressed as in Equations (3) and (4):(3)(4)

X = represents hydrogen ions, forming a number of pendant hydroxyl (OH) groups capable of forming hydrogen bonds. H + at position 2-, 3- and 6- in glucose are capable of forming donor bonds to grab metal ion.

Me = metal ion.

On the other hand, low chromium retention were only observed at pH = 2 due to metal ions being entrapped onto the bulky polymer volume at membrane surface [15]. At lower pH, nearly all chromium ions are recovered in the permeate stream. Retention of both chromium ions reached a plateau phase over pH 9. This was due to of the polymer site being filled with chromium ions, thus limiting the space for metal ions to be bounded when high pH is applied to the PEUF system. In addition to theoretical evidence, retention behavior of both chromium ions with unmodified starch could represent the complexation interaction through potentiometric studies of this chromium. Both chromium ions showed less significant adsorptive mechanism occurring at low pH range (between 2 to 6) where the behavior with or without the presence of chromium ions for complexation was similar, meaning that there was no complexation between chromium ions and unmodified starch.

#### Effect of pH on chromium ions retention using PEG

**Figure 8:**
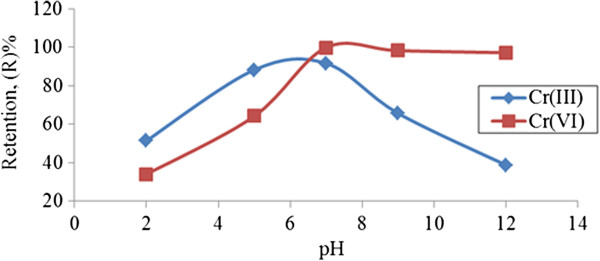
**Effect of pH on chromium ions retention using 1.0% (v/v) PEG.** (p = 1.5 bar, flowrate =115 cm/min).

Retention coefficient values at different pH ranges as revealed in (Figure 8) show the fluctuating behavior of both chromium ions species but the highest retention was obtained at pH 7.

Concentration of monovalent H^+^ ions is found to be higher, thereby contributing towards the low interaction behavior between metal ions-polymer at low pH values. At pH 3 and above, effective interaction of metal ions-polymer occurred particularly for trivalent metal ions as pH increased [15]. The plateau extends from pH 7 to 12, representing 99.8% retention of Cr (III) ions achieved at neutral pH region. This is in accordance with potentiometric titration studies for Cr (III) ions where more gaps appeared at pH 7, meaning that the complexation of Cr (III) and PEG occurred rapidly as pH increased to alkaline pH region. As there was no complexation between Cr (III)-PEG, retention dropped. For Cr (III) ions, the highest retention was probably due to Cr (III) attracted to the surface of nonionic PEG by physical adsorption.

PEG provided the available space to be penetrated by Cr (III) ions at natural pH where cationic species of Cr (III) ions dominate. Most probably the major surface charges of PEG are filled with negative charges, capable of adsorbing cation charges of Cr (III). As well, the occurrence of metal hydroxide precipitation is possible at neutral or alkaline pH values and is related to results found by Arthanareeswaran et al. [16]. Based on DOE discharge standards B for industrial effluent limits, both chromium ions species met the requirements which are 1.0 mg/l and 0.05 mg/l for Cr (III) and Cr (VI) [14] respectively, by applying PEG as the binding reagent at selected working conditions and pH 7 in the UF process.

This is contrary to Cr (VI) where the retention coefficient dropped as pH increased to alkaline pH region. Retention behavior of Cr (VI) was related to potentiometric studies of Cr (VI)-PEG where effective adsorptive mechanism occurred at pH 7-12 with the appearance of more gaps. As with the presence of Cr (VI) ions, the reactions slowly indicate complexation of Cr (VI)-PEG compared to neutralization by PEG alone.

### Effect of pH on chromium ions flux by PEUF

#### Effect of pH on chromium ions flux using unmodified starch

**Figure 9:**
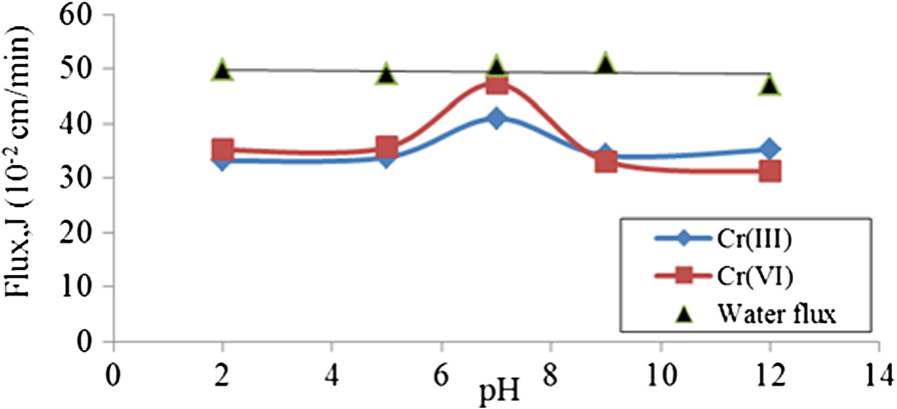
Permeate water flux, and chromium ions using 0.05% (w/v) unmodified starch at different pH values.

The permeate fluxes of 10 mg/l of both chromium ions at different pH ranges are plotted as shown in (Figure 9). As can be observed, there are no significant changes (<5 × 10^-2^ cm.min) in permeate flux for both chromium species as pH increases to pH 12. The shear force which formed because of the recirculation of feed solutions at lower polymer concentration is prevented since there is a possibility for polymer agglomeration on the membrane pores to take place. Only a slight increase on flux was obtained at pH 7.This was to be expected since gelatinization behavior of starch could negatively influence flux in PEUF studies. Results obtained at higher polymer concentrations in the study may not be valid in this UF study [5]. For Cr (VI), slight change to flux efficiency for all pH ranges was tested but there was no significant effect on Cr (III).

#### Effect of pH on chromium ions flux using PEG

**Figure 10:**
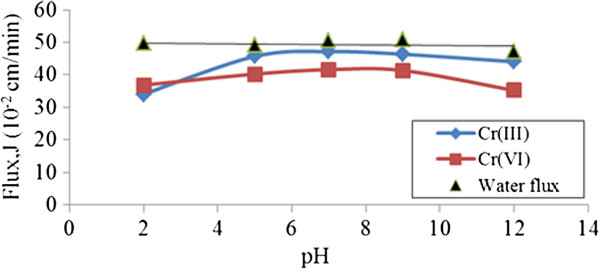
**Permeate water flux, and chromium ions using 1.0% (v/v) PEG at different pH values.**

(Figure 10) shows permeate water flux for both chromium ions using PEG polymer at pH varying between pH 2 to pH 12. It can be observed that flux could reach plateau phase at pH over 5 for both chromium ions. At very acidic pH regions, permeate flux of Cr (III) ions are obtained at only 34% at a constant PEG concentration of 1.0% (v/v).Changes in polymer molecular structure with pH is able to provoke the blockage of membrane pores at low pH range which influences the decrease on permeate fluxes [17].

For Cr (VI) ions, permeate flux was low at too acidic pH levels but gradually increased until pH 7 then slightly decreased at extreme pH 12 due to metal hydroxide precipitation [18]. The lower flux at pH 2 (≅36 cm^3^ cm^-2^ min^-1^) was probably due to the membrane suffering from a serious fouling phenomenon at high pH values [19]. In this study, the partial complexation of Cr (VI)–PEG complex cause the retention values to be low due to the failure of this macromolecular to be completely retained on the membrane.

### Effect of polymer concentrations on chromium ions retention by PEUF

#### Effect of polymer concentrations on chromium ions retention by unmodified starch

**Figure 11:**
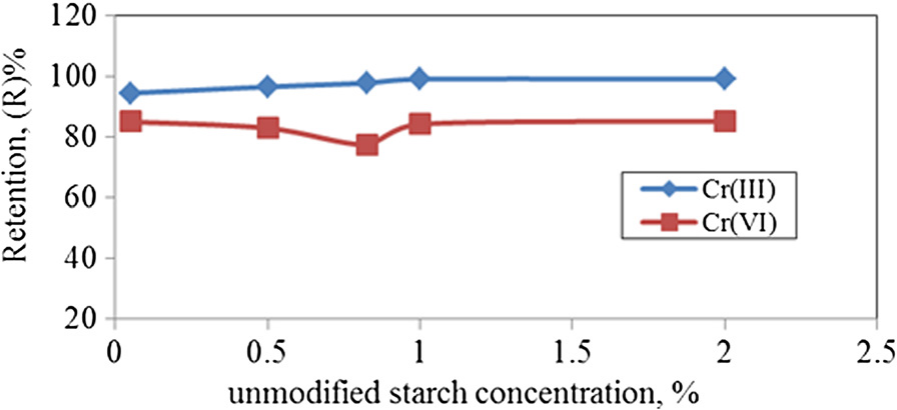
**Effect of unmodified starch concentrations on chromium ions retention.** (p = 1.5 bar, flowrate = 115 cm/min, pH = 7).

(Figure 11) shows the retention coefficients and selectivity for chromium ions removal at different unmodified starch concentrations. Retention coefficients of both Cr (III) and Cr (VI) could achieve about 95% and 85%, respectively by increasing starch concentration to 0.05%-2.5%. This is due to an increase of polymeric domains with local high and nearly constant ligand concentration which corresponds to an increase in the number of unmodified starch chains [15]. The difference caused by applying low or high polymer concentration results has little effect on retention. Since unmodified starch was expected to form gelatinization behavior that may interfere in the UF process, low concentration of unmodified starch is preferable.

Increase of unmodified starch concentration results in slight increase upon retention. When a too high concentration of starch was applied, it could be observed that complexation of both chromium ions to unmodified starch seems like stagnant bonding. Hence the bonding behavior of metal-polymer performed is not too complex to be retained on the membrane, thus resulting in retention coefficient reaching the plateau phase. This behavior has little effect on Cr (VI) retention when unmodified starch concentration increased over 0.5% of concentration, whereby metal-polymer became more complex at this stage.

#### Effect of polymer concentrations on chromium ions retention by PEG

**Figure 12:**
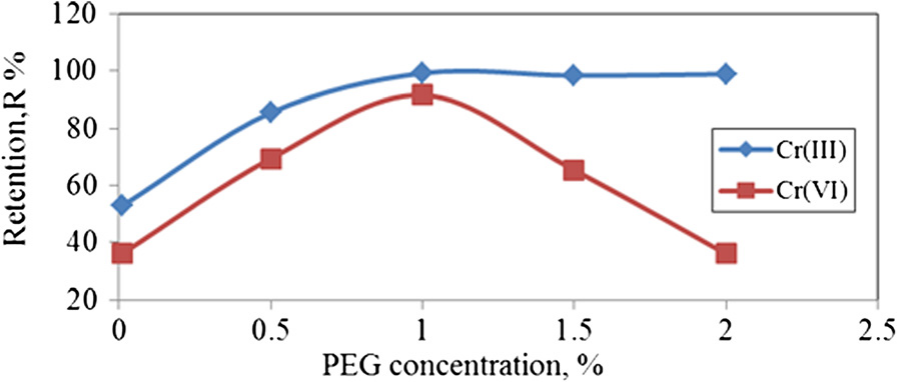
**Effect of PEG concentrations on chromium ions retention.** (p =1.5 bar, flowrate = 115 cm/min, pH = 7).

(Figure 12) shows the effect of PEG concentrations on both chromium ion species. Cr (III) shows an increase of retention at initial PEG concentration, and then stabilizing as 1.0% of PEG concentration is obtained. Constant retention of Cr (III) ions was due to the most favorable sites of polymer being fully filled by Cr (III) ions to form macromolecules as these are easier to be retained on membrane.

At certain optimum dosage of polymer, Cr (VI) seems unable to bind completely after an increase of PEG dosage up to 1.5%. This behavior may be explained by metal ions being trapped in polymer chains where the concentration of ligands is locally higher in the polymer domain. It is due to higher interaction among the backbone segments is allowed and adopting preferentially a coiled globule-like conformation hence, resulting in reduced viscosity [20] as PEG dosage increases. As feed solutions increase its concentration up to 2%, higher resistance due to influence of an increase of solute concentration in stagnant boundary layer on the membrane results, thus negatively affecting the retention coefficients [21].

#### Effect of metal ions feed concentrations on chromium ions retention by PEUF

Besides pH as a major fundamental factor, metal ions feed concentration also contributed towards efficiency of chromium ions removal in PEUF system. It should be noted that this parameter is important as it shows a tendency for chemical interaction towards certain metal cations and their ability towards complex metal-polymer [18,22].

#### Effect of metal ions feed concentrations on chromium ions retention by unmodified starch and PEG

(Table 1) shows the effects of chromium feed concentrations on retention coefficients by applying 0.05% unmodified starch concentration and 1.0% of PEG concentration.

**Table 1:** Effect of chromium feed concentrations on retention using selected polymers

**Metal ions**	**% Retention of Cr (III)**		**% Retention of Cr (VI)**	
**Concentration (mg/l)**	**Unmodified starch**	**PEG**	**Unmodified starch**	**PEG**
10	94.499	91.589	84.988	99.229
20	93.235	95.343	80.227	68.996
30	89.443	97.229	76.375	36.109
40	90.167	97.893	69.224	35.451
50	86.225	96.759	55.554	35.773

(unmodified starch concentration = 0.05% (w/v), PEG concentration = 1.0% (v/v), p = 1.5 bar, flowrate = 115 cm/min, pH = 7).

For retention coefficient employed by unmodified starch as water soluble polymer, Cr (III) ions obtained high retention at initial tested metal ion concentrations then decreased probably due to Cr (III) ions being partially bound to unmodified starch surface; in consequence, the decrease in retention coefficient represents at entire of tested metal ions concentrations.

For Cr (VI) ions, retention declined steadily when metal concentrations of increased 10 mg/l to 50 mg/l, which most probably causes an increase in ionic strength. Thus the retention affinity towards polymer was demolished because of an increase of dissociation constant [15].

For retention of both chromium ions by employing 1.0% PEG, pattern of retention coefficients performance is similar to retention using 0.05% of unmodified starch. By employed 1.0% of PEG, Cr (III) ions demonstrated less significant on retention for the effect of feed metal ions concentrations meaning only little changes of retention coefficient represents. The pattern is different for Cr (VI) ion retention as Cr (VI) ions decreased drastically as feed metal ions concentration increased from 10 mg/l to 30 mg/l and then reached a plateau phase at 40 mg/l and above. It could be expected that by applying low metal ions concentration, high retention of chromium ions could be achieved rather than applying high metal ions concentrations, which metal ion being partially bound to polymer, hence decreasing the retention coefficient [18].

#### Effect of metal ions feed concentrations on chromium ions flux by PEUF

Metal ions concentration exerts an influence on permeate flux of two chromium species in this PEUF study. This behavior is in contrast with pH, where less effect is found on flux. On the other hand, it has to be noted that explanation of metal ions concentration on permeate flux is also limited to PEUF studies. Given these circumstances, it will be interesting to explain this phenomenon in this paper.

#### Effect of metal ions feed concentrations on chromium ions flux using unmodified starch

During the optimization of the process, flux becomes one of the fundamental parameters in the PEUF study. Lower filtration area is required to process the desired amount of solution as higher permeate flux towards membrane is achieved.

(Table 2) shows the effect of different metal ions concentration values on the retention of Cr (III) and Cr (VI) from aqueous solutions by employing 0.05% of unmodified starch and 1.0% of PEG. Lower concentrations of chromium ions, 10 mg/l used in the UF process, most possibly provide favourable sites for both chromium ions to bond to unmodified starch, which has the potential to form macromolecular complexes. In contrast, using high metal ions concentrations will not assure higher flux efficiency of metal ions in the PEUF system.

**Table 2:** Effect of feed metal ions concentrations on chromium ions flux

**Metal ions concentration (mg/l)**	**Flux of Cr (III)**		**Flux of Cr (VI)**		**Water Flux (10**^**-2**^ **cm/min)**
	**(10**^**-2**^ **cm/min)**		**(10**^**-2**^ **cm/min)**		
	**Unmodified starch**	**PEG**	**Unmodified starch**	**PEG**	
10	39.846	38.167	39.846	19.551	49.712
20	34.221	34.671	39.987	24.561	49.034
30	36.113	41.621	36.113	28.643	50.641
40	32.114	39.874	32.114	24.231	50.981
50	30.278	42.116	25.113	18.492	47.181

(unmodified starch concentration = 0.05% (w/v), PEG concentration =1.0% (v/v), p = 1.5 bar, flowrate = 115 ml/min, pH = 7).

For results obtained on chromium ions flux by employing PEG, Cr (III) flux was obtained to be almost fluctuated by feed metal ions concentrations tested from 10 mg/l to 50 mg/l. Contrary to Cr (VI), the highest tendency to form metal ions complexes with PEG can be clearly observed at 30 mg/l, hence the highest permeate flux obtained is 28.643 × 10^-2^ cm/min. The difference in flux efficiency of Cr (VI) ions at 10 mg/l and 30 mg/l is less than 10 × 10^-2^ cm/min, hence 10 mg/l could still be applied as selected metal ions feed concentration in the UF system. On the other hand, as Cr (VI) ions increase to 50 mg/l, the ability to form macromolecules declined, hence negatively influencing flux efficiency. Consequently, complex molecule between metal-polymer cannot be completely retained on the membrane surface [18].

## Conclusions

Throughout this study, it was shown that complexation-ultrafiltration process has a great potential for chromium ions removal by engaging with two valuable polymers such as unmodified starch and PEG from synthetic aqueous solutions. A novel water-soluble polymer, unmodified starch was successfully able to remove Cr (III), and Cr (VI) ions were removed by the commonly used polymer, PEG at certain working conditions, especially at pH 7.

On the other hand, pH has been found to be a major factor in overcoming retention for Cr (III) and Cr (VI) ions species. A high retention of about 99.3% is shown by Cr (III) ions whenever pH increases to alkaline range for both tested polymers. This is in contrast to Cr (VI) where retention slightly decreased when applied with unmodified starch; a slight decay occurred as pH rose up to pH 12. The most interesting results were found with parameters of feed metal ions concentration. Retention coefficients approached 90% for both tested polymers at 10 mg/l. Permeate flux results obtained showed different behavior for Cr (III) and Cr (VI) at each feed metal ions concentration tested in this study. The effect of unmodified starch and PEG concentration on retention was also investigated. More convincing results were obtained on the removal of chromium ions where high impact of Cr (III) ions separations was achieved at 0.05% concentration of unmodified starch and 1% of PEG. The behavior of Cr (VI) was similar to Cr (III) but retentions were diminished when high concentration of PEG was applied.

Unmodified starch employed as water soluble polymer has the ability to adsorb metal cation. Additional chemicals or molecular structure readjustment is not required in correspond to less toxicity in consequence, unmodified starch is applied as polymer in PEUF system. The ability of metal ions retention by unmodified starch is high dependent on granular structure and not the type of starch. In consequences, it has a great influence on adsorptive mechanisms (probably caused by physical mechanism and high possibilities to occur chemically). The most efficient result is found in removal of Cr (VI) ions from aqueous solution by unmodified starch because Cr (VI) could be removed without reducing to Cr (III) and in consequence, could avoid precipitation during ultrafiltration process.

## Abbreviations

MWCO: Molecular weight cut off (Da); PEG: Polyethyle glycol; R: Metal retention coefficient; T: Temperature (°C); ∆P: Transmembrane pressure (bar); A: Effective membrane area (cm^2^); ∆V: Permeate volume (cm^3^); ∆t: Time (min); Cp: Concentration of metal ion in permeate (mg/l); Cf: Concentration of metal ion in feed (mg/l); C: Metal ion concentration (%); [Me]: Metal concentration (mol L^-1^); n: Coordination index of ligand L with metal Me; x: Value number of metal molecule coordination; PEUF: Polymer Enhanced Ultrafiltration; J: Permeate flux (cm/min).

## Competing interests

All authors declare that they have no competing interest.

## Authors’ contributions

NHB was the main investigator, designed and performed the study and drafted the manuscript. NMNS supervised the study. MKA were advisors of the study. NMNS and MKA also helped in the statistical analysis. All authors read and approved the final manuscript.
